# I. Mediastinal Abscess in a Cow Simulating Traumatic Pericarditis. II. Which Testicle Is Most Frequently Displaced in Cryptorchidism?

**Published:** 1898-02

**Authors:** S. J. J. Harger

**Affiliations:** Philadelphia, Pa.


### I. MEDIASTINAL ABSCESS IN A COW SIMULATING TRAUMATIC PERICARDITIS. II. WHICH TESTICLE IS MOST FREQUENTLY DISPLACED IN CRYPTORCHIDISM?

By S. J. J. Harger, V.M.D.,
PHILADELPHIA, PA.

I. The animal, a Jersey cow, had not been feeding as usual for about two weeks, when the services of a veterinarian, Dr. H. E. Wand, of Newtown, Pa., were requested. I am not in possession of the clinical details, but the symptoms so closely resembled traumatic pericarditis that a diagnosis of this disease was made and the slaughter of the animal recommended.

The autopsy revealed a few tubercles in the liver and on the peritoneum. In the lower portion of the posterior mediastinum was found a voluminous abscess with the following characteristics: Volume equal to that of the heart and oval in shape; the abscess was composed of a capsule of connective tissue about a quarter of an inch in thickness and easily enucleated from the surrounding tissue; the internal surface was smooth—a condition very much similar to that found in old cysts. The contents consisted of a thick, creamy pus with a fetid odor. The anterior surface of the abscess pressing against the pericardium caused a localized pericarditis, with adhesion, not only of the visceral and parietal layers, but the cicatricial tissue passed directly from the abscess-capsule into the heart-muscle and formed a firm union between the two. The pressure of the abscess against the left ventricle was so great that the latter, and in fact the entire heart was atrophied and showed a marked cavity on its posterior border, caused by atrophy from the pressure. A nail was found in the reticulum, but there was no fistulous tract leading in the chest-cavity, and there was only a small quantity of fluid in the pericardial sac which showed no signs of generalized inflammation.

The proximity of the abscess to the heart explains the similarity of the symptoms with those of pericarditis; the area of dulness on percussion was increased, although the heart-sounds on auscultation should have been less altered. This case shows an interesting fact in that, although the heart was the seat of and surrounded by marked pathologic alterations, the animal showed no outward signs of disease for a long time, and, reasoning from the thorough organization of the abscess-wall and its adhesions, the lesion must have been old. This characteristic is seen in lesions of many of the vital organs in ruminants, which may attain a remarkably advanced state before being detected.

II. This question is frequently asked. In monorchids, it is, according to some, the left, and according to others, the right testicle that has failed to reach the scrotum. There are, to the writer's knowledge, no special anatomic or physiologic peculiarities why one testicle more than the other should be more susceptible of being arrested in its descent from the sublumbar to the inguinal region. The number of cases has not been recorded, but the right testicle, in his experience, was by far the one that was most frequently displaced, and in the majority of cases the position of the testicle was abdominal, although most surgeons claim to find it in the inguinal canal.

Degive (of Belgium), Donald, Cadiot, and Trasbot found the left testicle in monorchids most often displaced, while to Haelst, Hering, and Franck this anomaly occurred in most instances on the right side. Cabat, of the Toulouse school, found this abnormal situation of the testicle nineteen times on the left side and eighteen times on the right. This veterinarian also found the testicle in fifty-three cases as follows: eight, both testicles in the abdomen; thirty, only one in this situation; eight, in the inguinal canal. It would seem that, making the observation upon a large number of cases collected from different operators, the abnormal displacement of the testicles seems to be about equally distributed upon both sides.

